# Parental separation: a risk for the psychomotor development of children aged 28 to 32 months? A cross-sectional study

**DOI:** 10.1186/s12887-016-0621-y

**Published:** 2016-07-11

**Authors:** Nadine Kacenelenbogen, Michèle Dramaix-Wilmet, M. Schetgen, M. Roland, Isabelle Godin

**Affiliations:** Département de Médecine Générale, Université Libre de Bruxelles, Campus Facultaire Erasme, Route de Lennik 808/612, Bruxelles, 1070 Belgium; Centre de Recherche en Epidémiologie, Biostatistique et Recherche Clinique, Ecole de santé publique, Université Libre de Bruxelles, Campus Erasme CP598, Route de Lennik 808, Bruxelles, 1070 Belgium; Service d’Information Promotion Education Santé-SIPES, Ecole de santé publique, Université Libre de Bruxelles, Campus Erasme CP598, Route de Lennik 808, Brussels, B-1070 Belgium; Avenue Molière 179, Bruxelles, 1190 Belgium

**Keywords:** Psychomotor performance, Preschool children, Parental separation, Prevention and screening

## Abstract

**Background:**

In Western countries, about a quarter of children are affected by parental separation and a number of authors have previously investigated how familial structure impacts children’s health. The purpose of the work: to analyze the psychomotor development of children aged 28 to 32 months based on family structure (parents together or separated), independently of the influence of socio-economic environment that is well documented.

To analyse the psychomotor development of children younger than 3 years based on family structure (parents together or separated) independently of the influence of socio-economic environment that is well documented.

**Methods:**

Cross-sectional study by examination of 28 871 children as part of a free preventive medicine consultation. The data came from an assessment conducted 28 to 32 months after birth during which information was collected about the psychomotor development: to perform a standing jump, dress themselves, draw a vertical line and circle, use the “I” pronoun, build a three-word sentence, and say their first name

**Results:**

Ten percent of the children had separated parents. Compared to parents who were together, when adjusting for the socioeconomic environment, as well as all potential confounders, the adjusted odds ratios (ORs) (95 % confidence interval [CI]) for children with separated parents, in terms of their ability to perform a standing jump, dress themselves, and draw a vertical line and circle were respectively 0.9 (0.7-1.1), 1.1 (0.9-1.2), 1.3 (1.1-1.4) and 1.2 (1.1-1.4). The adjusted ORs (95 % CI) for children’s inability to say the “I” pronoun, build a three-word sentence, and say their first name were respectively 1.2 (1.1-1.3), 1.3 (1.2-1.5), and 1.2 (0.9-1.5).

**Conclusions:**

After adjusting for sociocultural factors and other potential confounders, we observed that the children exhibited slower progression in psychomotor development, especially in language and graphic abilities when their parents were separated. While the implications of our study are somewhat limited, they do provide us with the necessary arguments enabling us to set up a prospective cohort study. Such a study should be able to better assess the impact of parental separation on the child's development, confirming our preliminary results.

## Background

A 2011 study conducted in Canada revealed that about 20 % of <15-year-olds were living with only one parent, while approximately 26 % of young Americans were living with only one of their parents in 2014 [1, 2]. In Australia, 15 % of children aged 0 to 4 years old were living in single-parent families in 2012–2013 and 5 % in blended families [3]. The situation is similar in Europe. In Great-Britain, 20 % of families with children were single-parent families in 2001 [4], and in 2006 alone, half of the separated couples were associated with over 125,000 children aged 0 to 16 years [5]. In Belgium, 20 % of children aged 0 to 16 were living in a single-parent or blended family in 2002 [6] and from 2008 to 2010, 6.8 % of infants aged 7 to 11 months were living in the same situation in the French community of Belgium, this percentage increasing to almost 10 % when the infants reached 30 months old [7]. A number of authors have previously investigated how familial structure influences children’s health on a somatic, psychological, and behavioural level. An American study involving 102,000 families between 2002 and 2003 demonstrated that, regardless of socioeconomic status, young people of all ages who were not living with both of their parents developed, among other issues, more adjustment disorders and difficulties at school, requiring more specialized care than others [8]. An US cross-sectional study has revealed that parental separation, is associated with more behavioural development disorders in children under 6 years compared to observations for situations when the parental couple is intact [9]. In Belgium, family doctors have noticed problems linked to the medical follow-up of children, as well as somatic, behavioural, and educational impacts, as a result of parental separations [10]. While there have been some studies establishing a significant link between parental separation and lower cognitive development score in school aged children [11], there are, to the best of our knowledge, only a few studies on this subject concerning preschool-age infants. The main objective of this study was therefore to assess the potential association between parental separation and the psychomotor development of children aged 28 to 32 months. The secondary objective was to identify the other factors related to the children’s delayed development at this age for potential applications in first-line treatment.

## Methods

### Study population

In the French community of Belgium, the *Office de la Naissance et de l’Enfance* ([ONE], Office of Birth and Childhood)offers a free preventive check-up program from pregnancy up to the child’s 6th year, compiling all the data in a computerized database. The data is collected for very young infants at five points in time: at birth in the maternity hospital, after discharge from hospital, between 7 and 11 months of age, between 16 and 20 months, and between 28 and 32 months. For each point of this check-up program, a data collection sheet is completed by a nurse, midwife, social worker, paediatrician or family doctor specifically trained for this task. Once completed, these results are anonymised and encoded in the central database. This system is in place for evaluation purposes and facilitates changes in policy concerning the areas of perinatal and early childhood social medicine. It should be noted that the data collection files pertaining to the three screening age groups were independent from each other, which forced us to employ a transversal analysis approach for our research. The research protocol was approved by the local ethics committee (ERASME hospital; medical board’s approval number: OM 021) on January 24, 2012 under the following reference: P2012/026.

We analysed the data of 30,769 children entered into the ONE database between 2006 and 2012 who had undergone a preventive health assessment 28 to 32 months after birth.

### Assessment of main exposure

Family structure was classified into the following seven categories: parents together, parents separated, the child only sees one parent, blended family, the child is in a children's home/foster home, other situations (living with grandparents, other family members), and unknown. Considering our definition of parental separation, i.e., parents not living together under the same roof, it should be noted that our analyses included only non-separated and separated parents (*n* = 28,871), meaning children who only saw one parent or were living in blended families falling under the second category.

### Assessment of other covariates

The other independent variables included in the analyses were the age of the mothers at childbirth and their level of education. Maternal age was categorized by separating very young mothers (<18) from older mothers (≥38, the age at which amniocentesis is systematically recommended). For the analyses, educational level was grouped into three categories (Table 1). For the variable concerning the language spoken at home, we merged the two different wordings used for the 2006–2009 and 2010–2012 periods so as to create a single variable reporting whether French was the child’s mother tongue or not. For analyzing variables concerning psychomotor development, we took into account the following additional parameters as potential influent factors: child’s gender, age in months in three categories (<27, 28–32, ≥33), birth weight (<2500 g vs. ≥2500 g), and body mass index (BMI) at the time of assessment. Hearing impairment was also screened for (whispered voice test) and data was collected on history of transtympanic drain and attendance at a day nursery or not between 2010 and 2012. BMI was calculated based on height and weight using the standard formula (weight in kg/height in m^2^). Based on the 2006 World Health Organization (WHO) growth standards, we determined whether the child exhibited a normal BMI-for-age (≥3rd percentile and ≤97th percentile) or not (<3rd percentile or >97th percentile). The “unknown” category answers were excluded from the analyses due to our previous observations that the distribution of socioeconomic status variables did not significantly differ among this group.

**Table 1:** Description of the study population

Variable (*without *unknown data*)	%
Description of mother’s characteristics	
Mother’s age at childbirth (years) *n* = 28,126	
<18	0.9
18/30	59.1
31/37	33.0
≥38	7.0
Mother’s level of education *n* = 24,211	
<Upper secondary education	22.0
Upper secondary education completed	31.0
Higher education/academic or not	47.0
Mother’s native language *n* = 27,979 (2006–12)	
French	77.4
Other language	22.6
Family structure *n* = 28,871	
Separated parents/sees only one parent/blended family	9.8
Parents living together	90.2
Description of child’s characteristics	
Sex *n* = 28,629	
Male	51.3
Female	48.7
Children’s age (months) *n* = 28,819	
≤27	7.1
28–32	89.2
≥33	3.7
Birth weight (g) *n* = 28,862	
<2500	6.9
≥2500	93.1
BMI (Kg/m^2^) at the time of health check *n* = 27,355	
P3-P97	92.0
>p97	6.2
<p3	1.8
Normal hearing *n* = 23,948	
Yes	97.5
No	2.6
Transtympanic drains *n* = 2,662	
Yes	5.0
No	95.0
Day nursery attendance *n* = 11,798 (2010–12)	
Yes	55.8
No	44.2

### Outcome ascertainment

The dependent binary variables considered based on our research question were the children’s ability to perform a standing jump, dress themselves (except shoes and buttons), draw a vertical line and circle, use the “I” pronoun, build a three-word sentence, and say their first name. These tests feature as part of the Denver Developmental Screening Test (DDST) [12] For the variables concerning the children’s ability to dress themselves and use the “I” pronoun, the analysis was only conducted for data from the 2006–2009 period. For the variable describing the children’s ability to say their first name, only 2010–2012 data was used. We thus analyzed the entire cohort according to the availability of variables in the different periods (2006–2012, 2006–2009, and 2010–2012). This process of restricting the analysis to one of the two periods was not problematic given the large sample size.

### Statistical analysis

We conducted the Chi-squared test, deriving the odds ratios (ORs), along with their confidence intervals at 95 % (95 % CI), in order to compare the two groups of children aged 28 to 32 months, namely those exposed to parental separation versus those not. Multivariate analyses of binary logistic regression were used to adjust the effect of exposure. The models were designed using a backward selection method for potential confounders and the parental situation variable was automatically included in the models. The interactions between this variable and other predictors were tested, with the analysis by child gender proving significant for the children’s ability to say their first name and draw a circle. We performed the Hosmer–Lemeshow test in order to confirm the models’ suitability. The absence of collinearity between the predictors included in the model was verified by means of variance inflation factors (VIFs). All analyses were conducted using the STATA 12.0 software (http://www.stata.com).

## Results

At birth, 60 % of mothers were aged between 18 and 30 years, while 1 % were under 17. Approximately 46 % had obtained a higher education diploma, whereas 22 % had not finished secondary education, and French was not the mother tongue in 22 % of the families (Table 1). In our population, approaching 10 % of children had separated parents, lived in blended families or saw only one of their parents (Table 1). Our sample comprised slightly more boys than girls and the proportion of children with a low birth weight (<2500 g) amounted to 7 %. On clinical work up, 2 and 6 % of the children exhibited a BMI inferior to the 3rd percentile and superior to the 97th, respectively, and 3 % exhibited hearing impairment. We also found that 55 % attended a day nursery (Table 1). From 2006 to 2012, 22 % of the children affected by parental separation were unable to draw a vertical line, as compared to nearly 17 % of those whose parents were together (*p* <0.001) (Table 2). For the same period, 30 % of those affected by parental separation were unable to draw a circle, compared to slightly less than 25 % of those whose parental couple was intact (*p* <0.001) (Table 2). Between 2006 and 2009, 52 % of the children affected by separation did not use the “I” pronoun, as compared to 46 % of those whose parents were together (*p* <0.001). Between 2010 and 2012, 20 % of the children whose parents were separated were unable to say their first name, versus 15 % of those with parents still together (*p* <0.001) (Table 2). Between 2006 and 2012, approximately 25 % and 18 % of the children in separated-parent and together-parent families, respectively, were unable to build a three-word sentence (*p* <0.001) (Table 2). As for the children’s ability to perform a standing jump and dress themselves, however, no significant difference was observed according to family structure (Table 2). After adjusting for sociocultural factors and other potential confounders provided by the database, parental separation was found to still significantly correlate to the children’s graphic ability (vertical line and circle) and language acquisition (“I” and three-word sentence) variables (Table 3). The adjusted ORs were generally slightly lower than the crude ORs (Table 3) and, once adjusted, the OR for the children’s ability to say their first name was no longer significant (Table 3). For this variable, we observed a significant interaction between the child’s gender and the family structure. In boys, the adjusted OR regarding the children’s ability to say their first name in cases involving separated parents was 1.5 (95 % CI: 1.2-2.0), whereas the association with family structure was not found to be significant in girls, with an OR of approximately 1 (Table 4). A similar correlation was observed relating to the inability to draw a circle. In boys, the adjusted OR was 1.4 (95 % CI: 1.2-1.7) in cases involving parental separation, whereas the association was non-significant in girls. Hearing impairment was the most strongly associated factor with the different variables corresponding to the children’s psychomotor development, with adjusted ORs ranging from 2 to 8 (Table 3). Male gender was also associated with an increased risk of slowed acquisition of skills, as was the factor of the mother’s young age at childbirth and low level of education, as well as a child’s low birth weight and abnormal BMI (<3rd percentile or >97th percentile) at assessment time (Table 3). Unsurprisingly, the younger the child, the less successful he was in the different tests (Table 3). Moreover, for the 2010–2012 period, not attending a day nursery was associated with children’s inability to build a three-word sentence and say their first name, with adjusted ORs of 1.6 (95 % CI:1.4-1.9) and 1.8 (95 % CI: 1.5-2.0), respectively (Table 3). It should also be noted that, when French was not the children’s mother tongue, they were less often able to say their first name (1.3; 95 % CI: 1.1-1.5), yet were more likely able to use the “I” pronoun in their language and dress themselves, with an adjusted OR of 0.8 (95 % CI: 0.7-0.9) in both cases (Table 3).

**Table 2:** Psychomotor development of children aged 28 to 32 months

Variables describing the psychomotor development	Total	Parents together	Separated parents	p
Children cannot perform a standing jump (2006–12)	(*n* = 22082)	(*n* = 19916)	(*n* = 2165)	0.6
%	7.4	7.5	7.2	
Crude OR (95 % CI)		1	1.0 (0.8-1.1)	
Children cannot draw a vertical line (2006–12)	(*n* = 17857)	(*n* = 15120)	(*n* = 1736)	<0.001
%	17.7	17.3	21.9	
Crude OR (95 % CI)		1	1.3 (1.2-1.4)	
Children cannot draw a circle (2006–12)	(*n* = 16193)	(*n* = 14623)	(*n* = 1569)	<0.001
%	24.9	24.4	29.9	
Crude OR (95 % CI)		1	1.3 (1.2-1.4)	
Children cannot dress themselves (2010–12)	(*n* = 9481)	(*n* = 8495)	(*n* = 986)	0.1
%	34.7	34.5	36.5	
Crude OR (95 % CI)		1	1.1 (0.97-1.2)	
Children cannot use the “I” pronoun (2006–09)	(*n* = 8021)	(*n* = 7197)	(*n* = 823)	<0.001
%	46.8	46.1	52.4	
Crude OR (95 % CI)		1	1.3 (1.2-1.4)	
Children cannot build a 3-word sentence (2006–12)	(*n* = 22522)	(*n* = 20335)	(*n* = 2186)	<0.001
%	18.1	17.5	24.7	
Crude OR (95 % CI)		1	1.5 (1.3-1.6)	
Children cannot say their first name (2010–12)	(*n* = 9535)	(*n* = 8672)	(*n* = 862)	<0.001
%	16.0	15.5	20.4	
Crude OR (95 % CI)		1	1.4 (1.2-1.6)

**Table 3:** Psychomotor development of children aged 28 to 32 months: adjusted Ors

	Children cannot perform a standing jump	Children cannot draw a vertical line	Children cannot draw a circle	Children cannot dress themselves	Children cannot use the “I” pronoun	Children cannot build a 3-word sentence	Children cannot build a 3-word sentence	Children cannot say their first name
	(2006–12)	(2006–12)	(2006–12)	(2006–09)	(2006–09)	(2006–12)	(2010–12)	(2010–12)
Variable	OR	OR	OR	OR	OR	OR	OR	OR
	(95 % CI)	(95 % CI)	(95 % CI)	(95 % CI)	(95 % CI)	(95 % CI)	(95 % CI)	(95 % CI)
Family structure								
Parents together	1	1	1	1	1	1	1	1
Parents separated	0.9 (0.7-1.1)	1.3 (1.1-1.5)	1.2 (1.1-1.4)	1.1 (1.0-1.2)	1.2 (1.1-1.3)	1.3 ((1.1-1.5)	1.4 (1.1-1.7)	1.2 (1.0-1.5)
p	0.5	<0.001	<0.001	0.2	0.003	<0.001	0.002	0.07
Mother’s level of education								
Higher education	1	1	1		1	1	1	1
Complete upper secondary education	1.1 (0.9-1.2)	1.3 (1.2-1.5)	1.2 (1.1-1.3)		1.2 (1.1-1.3)	1.6 (1.5-1.8)	1.3 (1.1-1.6)	1.6 (1.4-1.9)
<Upper secondary education	1.5 (1.3-1.7)	1.7 (1.5-1.9)	1.6 (1.4-1.7)		1.3 (1.1-1.4)	2.6 (2.3-2.9)	2.0 (1.6-2.4)	2.2 (1.8-2.6)
p	<0.001	<0.001	0.001		<0.001	<0.001	<0.001	<0.001
Mother’s age at childbirth								
< 18 years			1.2 (0.8-1.7)			1.2 (0.8-1.7)	1.8 (1.1-3.1)	2.2 (1.3-3.7)
18/30 years			1			1	1	1
31/37 years			1.1 (1.0-1.2)			1.1 (1.0-1.2)	1.2 (1.0-1.4)	1.1 (0.9-1.3)
38 years and over			1.3 (1.1-1.5)			1.3 (1.1-1.5)	1.5 (1.2-1.9)	1.5 (1.1-1.9)
p			0.004			0.01	<0.001	0.001
Mother’s native language French								
Yes				1	1			1
No				0.8 (0.7-0.9)	0.8 (0.7-0.9)			1.3 (1.1-1.5)
p				0.02	<0.001			<0.001
Day nursery attendance								
Yes							1	1
No							1.6 (1.4-1.9)	1.8 (1.5-2.0)
P							<0.001	<0.001
Birth weight								
≥2500 g	1	1		1	1	1	1	
<2500 g	1.6 (1.3-1.9)	1.2 (1.0-1.4)		1.3 (1.1-1.5)	1.4 (1.2-1.6)	1.4 (1.2-1.6)	1.3 (1.0-1.7)	
p	<0.001	0.01		0.003	<0.001	<0.001	0.04	
Gender								
Female	1	1	1	1	1	1	1	1
Male	1.2 (1.1-1.4)	1.6 (1.5-1.7)	1.6 (1.5-1.8)	2.1 (1.9-2.2)	1.5 (1.4-1.6)	2.0 (1.8-2.2)	2.0 (1.7-2.3)	1.8 (1.6-2.1)
p	<0.001	<0.001	<0.001	<0.001	<0.001	<0.001	<0.001	<0.001
Children’s age								
≥33 months	1		1	1	1	1	1	1
28-32 months	1.0 (0.8-1.4)		1.2 (1.0-1.5)	1.6 (1.3-2.0)	1.7 (1.4-2.1)	1.5 (1.2-1.9)	2.1 (1.4-3.3)	1.5 (1.0-2.2)
≤27 months	1.5 (1.1-2.0)		1.6 (1.3-2.1)	2.3 (1.7-3.0)	2.1 (1.7-2.7)	2.4 (1.9-3.2)	3.1 (1.9-5.1)	2.4 (1.6-3.8)
p	<0.001		<0.001	<0.001	<0.001	<0.001	0.03	<0.001
Normal hearing								
Yes		1	1	1		1	1	1
No		2.9 (2.3-3.6)	2.3 (1.9-2.9)	1.9 (1.5-2.5)		8.1 (6.5-10.1)	6.6 (4.4-9.7)	7.4 (5.1-10.9)
p		<0.001	<0.001	<0.001		<0.001	<0.001	<0.001
Transtypanic drains								
Yes						1		
No						1.6 (1.4-1.9)		
p						<0.001		
Children’s BMI								
p3-p97				1				1
>p97				1.2 (1.0-1.4)				1.4 (1.0-1.8)
<p3				1.3 (1.0-1.8)				1.5 (0.9-2.3)
p				0.03				0.02

**Table 4:** Gender disparities

	Children cannot say their first name^a^		Children cannot draw a circle^b^	
Family structure	Female (*n* = 3510)	Male (*n* = 3577)	Female (*n* = 7999)	Male (*n* = 8174)
	adjusted OR	adjusted OR	adjusted OR	adjusted OR
	(95 % CI)	(95 % CI)	(95 % CI)	(95 % CI)
Parents together	1	1	1	1
Parents separated	0.9 (0.6-1.3)	1.5 (1.2-2.0)	1.0 (0.8-1.2)	1.4 (1.2-1.7)
p	0.6	<0.004	0.9	<0.001

*OR* odds ratio; *CI* confidence interval

^a^Adjusted OR for children’s age, birth weight, drain presence, body mass index, and hearing abilities, as well as for day nursery attendance, mother’s age at childbirth, and level of education

^b^Adjusted OR for children’s age, hearing abilities, and mother’s level of education

## Discussion

After adjusting for sociocultural factors and mother’s age at childbirth, along with other potential confounders like the children’s own characteristics, we observed that the children exhibited slower progression in psychomotor development, especially in language and graphic ability, when their parents were separated.

### Strengths and limitations of the study

Caution should be exercised in interpreting these results owing to the methods we employed. The cross-sectional nature of our study resulted in uncertainty with regard to time. Given that the development tests were carried out by the physician working for the ONE organisation, parental separation certainly occurred prior to the screening date. It must, however, be mentioned that the exact date of parental separation remains unknown to us. Of note is also that the data, such as parental education level or the child's family situation, was retrieved from the parent(s) that accompanied the child, which certainly constitutes a limitation to our research work. The children’s ability to perform a standing jump, dress themselves, and draw a vertical line and circle, as well as their language acquisition level, which was judged by their ability to build a three-word sentence, are all considered pertinent criteria for assessing their psychomotor development. These criteria are not only used in the DDST [12], but also in other screening tools adapted for children of preschool age, such as standard developmental milestones [13] and the Bayley Scales of Infant and Toddler Development [14]. The ONE opted for these tests due to their ease of use in first-line consultation. Studies have proven the DDST’s validity for detecting developmental delays on a large scale [15, 16] and it is regularly used as a reference tool [17]. Nevertheless, the child’s cooperation is required for performing these tests, which is not always easy to achieve during a preventive-medicine consultation of limited duration, thus accounting for the relatively high number of “uncompleted examinations” leading to observation loss. Still, the number of observations we achieved remained substantial, with approximately 20,000 examinations completed for the 2006–2012 period and approximately 10,000 for the 2006–2009 and 2010–12 periods. The analysis of the children who did not complete the tests revealed that the examination was more frequently interrupted when the mother had a low level of education or did not speak French as their mother tongue. The population studied between 2006 and 2012 represented, for each of the 7 studied years, approximately 5 % of the total population of that age in the French community of Belgium [18]. Our sample, however, is not entirely representative of the general population in terms of social level. For example, while slightly more than one-third of mothers had completed upper secondary education, as is the case in the general population, those with a higher education level were overrepresented in our study (46 % vs. 26 % in the general population), whereas mothers who had not completed secondary education were underrepresented (22 % vs.40 %) [19]. A possible explanation for this could be that the ONE preventive medicine program is wholly free of charge and accessible to all, with no exceptions, yet is not mandatory. Our study population was thus composed of families who made the voluntary choice of bringing their child to the ONE for preventive examinations, a choice found to be more frequently made in less impoverished families, which may have introduced a bias. Nevertheless, this bias was only of relative importance, since our study sought to compare children living with both parents to those not, which was still possible in our sample given that all sociocultural levels were sufficiently represented. Furthermore, our study hypothesis held that parental separation could present a risk factor for developmental delay, independent of social environment. Several studies have demonstrated that socioeconomic status is a major indicator of children’s neurocognitive performance and that there is a negative correlation between the progression of instability and the children’s optimal development [20, 21]. Yet as our study sample was composed of children from less underprivileged families, it could be suggested that, should our results feature a bias, it would weaken their significance. In addition, it should also be noted that there is no other database in Belgium that systematically collects the psychomotor development levels of children of this age in the general population.

### Comparison with other studies

Studies specifically assessing the possible association between parental situation (parents living together or not) and children’s psychomotor development are few and far between. One example is an American review, which aimed to assess the use of risk factors for the first-line detection of language delays in children of preschool age. In this study, while the family history of language delay, male gender, parental education level, child’s medical history since birth, and number of brothers and sisters were all mentioned as risk factors, neither the mother’s marital status nor the family structure (parents together or separated) were taken into account [22]. In contrast, a Brazilian study reported a significant adjusted association existing between developmental delay in children aged 0 to 6 years, on the one hand, and poverty, very young maternal age, and also lack of paternal support for the child’s education on the other [23]. More specifically, a longitudinal study conducted in New York involving 290 children aged between 24 and 36 months revealed paternal involvement to have a positive impact on language acquisition [24]. Another longitudinal study, conducted in Great Britain, also found that children aged 0 to 8 years who were exposed to parental separation produced poorer results for language, reading, writing, and drawing tests following adjustment for sociocultural environment [9]. In Belgium, there was also a cross-sectional study conducted in 7- to 11-month-old infants that observed a significant correlation between delayed psychomotor development and parental separation [25]. In this study, the variable pertaining to the child’s psychomotor development assessed whether there were at least two anomalies at the time the assessment was conducted, while taking into account the child’s age expressed in months as based on the neurological examination. In comparison with the child living together with its parents, a psychomotor developmental delay was observed more often in those whose parents were separated, with an adjusted OR of 1.3 (95 % CI: 1.1-1.6). Further contributing to this area of research, a Spanish prospective study involving 433 2-year-old children sought to determine the positive predictors of psychomotor development, among others, so as to create an evaluation scale that also integrates family context. These researchers identified the most potent indicators such as paternal involvement and infrequent or minor family conflicts, as well as low exposure to parental and child stress [26]. Certain literature reviews and qualitative studies have associated parental separation with the accumulation of risk factors for developmental delay, such as family conflicts, one parent’s loss of interest in the child’s education, and parental psychopathology [10, 27]. Several studies have, in fact, observed that maternal psychopathology [28, 29] may induce developmental disorders in children [30, 31]. Yet other studies, some prospective in nature [32], have demonstrated the positive impact of breastfeeding for at least 6 months on the psychomotor development of children during the first 5 years of life. Other studies have revealed a relationship between maternal marital status and breastfeeding [33], in particular including a cross-sectional study demonstrating a significant association between parental separation and specific parental behaviors, such as less frequent breastfeeding or earlier weaning [34]. This relationship between breastfeeding and the child’s development could at least be partly accounted for by the regulation mechanisms of cortisol metabolism and stress in children [35, 36]. Our other results are consistent with those described in the literature: children’s psychomotor development is impacted by socioeconomic status [21, 22], mother’s age at child birth, education level of the parents (especially the mother) [23, 37], child’s birth weight [31], and child’s hearing quality due to the involvement of the mirror neuron system [38, 39]. In line with the literature, our analyses also revealed that psychomotor development was systematically slower in boys [23]. Furthermore, we observed that boys were particularly sensitive to the family environment, notably regarding the ability to say their first name or draw a circle (Table 4). Similar results can be found in the literature, with a Swedish longitudinal study concluding that boys are more vulnerable to psychosocial difficulties when it comes to their acquisition of graphic skills and language [40]. We observed a significant correlation between abnormal BMI and the child’s psychomotor development for only two abilities: children’s ability to dress themselves and say their first name. As in our study, the literature does not report any significant association between motor skill acquisition and BMI [41, 42]. Some studies, however, have described a relationship between the behavioural and cognitive status in school-age children and a history of failure to thrive during early childhood [43, 44]. In our study, the children whose mother tongue was not French were more rapidly able to use the “I” pronoun in their language, though they took longer to say their first name. This finding is not, however, clearly supported in the literature. Finally, echoing the literature, our results revealed that children attending a day nursery were more rapidly able to say their first name or build a three-word sentence than others [45]

## Conclusions

Implications for care givers involved in the child’s development. Our study has confirmed the need for screening interventions focused on the psychomotor development of children of preschool age, namely regarding graphic skills and language acquisition. This screening is especially crucial in impoverished families, when the mother is very young or has a low education level, and when the child had a low birth weight, as well as in cases of child hearing impairment. In this regard, the proactivity of family doctors remains essential, given that almost all families in Belgium have an appointed family doctor typically consulted on average four times a year by 90 % of adults and 70 % of children. We also know that the poorer families are, the more often they go to see their general practitioner [46]. Our research exhibits several limitations, such as selection bias, transversal analysis methodology, most data being retrieved from the declarations of the parent accompanying the child with uncertainty about the exact date of parental separation, etc. However, it must be pointed out that our results reveal that, irrespective of the child’s birth weight, BMI, hearing quality, or cultural environment, and of the mother’s education level, along with all the potential related factors in terms of child development, children aged 28 to 32 months were shown to exhibit a slower acquisition of graphic and language skills when affected by parental separation than those living with an intact parental couple. Despite the low ORs with respect to the large sample size, parental separation concerns one-quarter of the country’s paediatric population [6], thus displaying a huge impact in terms of public health issues. Several studies have revealed that in comparison with other children, those exhibiting developmental delays at preschool age were more exposed during their lifetime to poorer health conditions, socio-economical problems, as well as social isolation. And in addition, early support for parents was shown to be efficacious in slowing down the aggravation of difficulties [47, 48]. The same type of conclusions can be drawn for children with so-called delay in language acquisition. Even if most of them will recover and be able to speak normally at the age of 5–7 years, they are meant to suffer more frequently from problems pertaining to the socio-affective domain [49]. Even though this study does not provide any explanation concerning the link between parental separation and developmental delay, it encourages us to revise our commonly-held assumption that the less-than-optimal development of children with separated parents is due solely to a more economically precarious environment. General practitioners do play a crucial role in both the prevention and detection of health risk in very young children. It therefore appears paramount that the primary care physician gathers information about the child's family environment.

In the event of parental separation, and regardless of socioeconomic situation, the family doctor should then be even more attentive with regard to the child’s psychomotor development, especially for boys. In Belgium, it is already advised that family doctors enquire about the quality of the partners' relationship every time they have contact with a family with a very young child or children [50]. Our results confirm the validity of this approach, which makes it possible to support couples with children of preschool age. It can be presumed that if the suggested recommendations are properly understood, i.e., applied in a kind and understanding manner without stigmatizing people or making them feeling guilty regarding the separation, then the benefits, however small, will outweigh the resulting risks. A possible hypothesis holds that parental separation could constitute a risk factor, among others (genetic, environmental, socioeconomic, somatic, parental psychopathology), of slower acquisition of motor and cognitive skills. In Belgium, this slower psychomotor development poses a problem from the very beginning of school attendance. Even in nursery schools, teaching does not take into account the children’s acquisitions, only their age, inducing an associated risk of progressively widening the gap and exacerbating inequality, whatever the cause [51]. Finally, it should be noted that the types of academic difficulties experienced by children with separated parents have been regularly described in the literature [52, 53].

## Abbreviations

BMI, Body mass index; DDST, Denver Developmental Screening Test; IC, Confidence interval; ONE, Office de la Naissance et de l’Enfance (Office of Birth and Childhood); OR, Odd Ratio
